# Inferring microbial interactions in thermophilic and mesophilic anaerobic digestion of hog waste

**DOI:** 10.1371/journal.pone.0181395

**Published:** 2017-07-21

**Authors:** Grace Tzun-Wen Shaw, An-Chi Liu, Chieh-Yin Weng, Chu-Yang Chou, Daryi Wang

**Affiliations:** 1 Biodiversity Research Center, Academia Sinica, Taipei, Taiwan; 2 Institute of Biological Chemistry, Academia Sinica, Taipei, Taiwan; 3 Department of Bio-Industrial Mechatronics Engineering, National Taiwan University, Taipei, Taiwan; Kyungpook National University, REPUBLIC OF KOREA

## Abstract

Anaerobic digestion (AnD) is a microbiological process that converts organic waste materials into biogas. Because of its high methane content, biogas is a combustible energy source and serves as an important environmental technology commonly used in the management of animal waste generated on large animal farms. Much work has been done on hardware design and process engineering for the generation of biogas. However, little is known about the complexity of the microbiology in this process. In particular, how microbes interact in the digester and eventually breakdown and convert organic matter into biogas is still regarded as a “black box.” We used 16S rRNA sequencing as a tool to study the microbial community in laboratory hog waste digesters under tightly controlled conditions, and systematically unraveled the distinct interaction networks of two microbial communities from mesophilic (MAnD) and thermophilic anaerobic digestion (TAnD). Under thermophilic conditions, the well-known association between hydrogen-producing bacteria, e.g., *Ruminococcaceae* and *Prevotellaceae*, and hydrotrophic methanogens, *Methanomicrobiaceae*, was reverse engineered by their interactive topological niches. The inferred interaction network provides a sketch enabling the determination of microbial interactive relationships that conventional strategy of finding differential taxa was hard to achieve. This research is still in its infancy, but it can help to depict the dynamics of microbial ecosystems and to lay the groundwork for understanding how microorganisms cohabit in the anaerobic digester.

## Introduction

Renewable energy has received great attention in recent years due to the shortage of fossil fuels and increasing atmospheric pollution and global warming caused by the burning of fossil fuels [1]. Biogas produced from the AnD of biomass or organic waste is renewable, environmentally friendly and associated with multiple benefits [2–5]. A balanced interaction between microbes in the bio-digestion chain is crucial for a stable and efficient gas production. With traditional culture-dependent techniques, researchers were able to identify the microbes responsible for specific metabolic processes in AnD [6]. However, most microbes in these systems are still uncultivatable, and the key species for efficient biogas production are not fully known. Moreover, a number of studies have shown that the roles and interactions of specific microorganisms within the biogas-producing communities are very complex [7–10]. An in-depth study to understand the interactive structure of the microbial community and its functional dynamics during different phases of AnD is absolutely necessary if we want to improve the process and performance of biogas production in a microbial digester.

A conventional way to decipher microbial interactions is performing co-culture experiments which construct an artificial community in a controlled condition [11, 12]. Although the co-culture strategy aimed to monitor population dynamics, the microbial abundance frequently shows the difference between structured and unstructured environments [12]. Another problem is that co-culture experiments routinely involve only a small number of microbial species. In a systematical way, one key method to improve understanding of microbial relations in AnD reactors is through network inference. In order to uncover the hidden patterns beyond the animal world, network-based approaches have been applied to investigate the interactions between animals. Network inference has been widely used to explore interactions between various entities [13, 14]. However, microbes are the most abundant and diverse organisms on earth, and microbial interactions, including mutualism, competition, parasitism and commensalism, are difficult to quantify as the underlying processes usually cannot be observed directly and are often too complex for laboratory experiments [15]. Fortunately, recent advances in high-throughput sequencing technology have made large scale surveys of microbial communities feasible. Metagenomic studies and network-based approaches have yielded detailed information on the composition of microbial communities, which in turn have paved the way to study the structure of microbial ecosystems and their dynamics [16–18].

There are several approaches that can be taken to construct a microbial network. One commonly applied method is the similarity-based network construction where the co-occurrences of two species over multiple time-series samples are measured to infer their interactions [16, 17, 19–21]. In such networks, nodes correspond to organisms and an edge between two nodes represents the significant relationship of two taxa across a set of time series samples. Although the advanced version of this approach [22–24] can identify pairwise relationships using a correlation estimation, it is still difficult to identify the direction and the strength of these relationships between microbes [15]. Recently, a number of methods employing mathematical and statistical models have been developed to model complex relationships (one species influencing multiple others). For example, some studies used nonparametric regression models to infer the dynamic relationships between three microbial populations [25, 26]. Recently, a new tool called MetaMIS (**Meta**genomic **M**icrobial **I**nteraction **S**imulators) that applies generalized Lotka-Volterra models (gLV) was released [27]. The gLV equations also known as the predator–prey equations have recently been adopted for the study of dynamic models for microbial communities [15, 28]. They are commonly used to model direct competition and trophic relationships between an arbitrary number of species. In a dynamic model of a microbial community from 16S rRNA amplicon data, the input data comprise the growth rates and relative abundance of the community members, which can either be obtained experimentally or can be estimated from time series data. According to our survey, gLV equations can be used to study many aspects of microbial interactions; some studies indicated that gLV equations can successfully predict microbiota temporal dynamics from the mouse intestine [28] or within a cheese-making environment [29]. On the other hand, some studies suggested that the distribution of interaction pairs obtained by a gLV dynamic model in an ecological system can be used to predict microbiota stability [30]. Another important application of this approach is in data simulation. Given a set of known interspecies interactions, the LV model can generate a simulated microbial community [30–32]. These applications make it a potential tool for future metagenome analysis.

AnD can be maintained at different temperature ranging from mesophilic (30–40^°^C) to thermophilic (55–65^°^C) temperature [33]. It is known that thermophilic temperatures lead to higher substrate degradation and biogas production. Several studies have demonstrated the different bacterial composition of mesophilic and thermophilic anaerobic digesters using swine manure [34, 35], however, limited information regarding the specific roles played by each microbe was revealed. In order to more closely investigate the interaction patterns in these two conditions and clarify the major microbes which potentially drive the changes of the communities, we collected 16S rRNA profiles from day 18 to day 60 (the start-up to steady-state stage in the swine manure digester) and analyzed the microbial interactions using MetaMIS [27]. We found that the microbial interaction network could serve to reinforce the understanding of microbial community and to systematize these pairwise interactive relations. The thermophilic interaction network showed a very different profile of influential microbial members than those of the mesophilic network. Through these network-based approaches, we have revealed the first information on the interactions of microbial communities in an anaerobic digester, which in turn will pave the way to study the structure and dynamics of the microbial ecosystems under these conditions.

## Materials and methods

### Anaerobic digester operation and sampling

To compare the microbiota composition in thermophilic (55^°^C, TAnD) and mesophilic (37^°^C, MAnD) AnD digesters, two bench-top CSTRs (completely stirred tank reactors), each with a working volume of 3 L, were set up for this study [36]. The digesters were operated at two different temperatures, mesophilic (37^°^C) and thermophilic (55^°^C), using 15 days hydraulic retention time (HRT), and 5% total solids (TS) without pH control. All tests were operated at the theoretical steady state in each reactor with a semi-continuous feeding mode. Effluents were collected and influents were added every day. Manure from hogs, the growing pigs, from the same farm was used as the primary substrate to establish the baseline information of the operational parameters, including temperatures, TS concentrations and HRT. We received approval from the farm owners to collect the concentrated pig manure from her pig farm, called DaXi pig farm, in Miaoli County, Taiwan. These manure samples were kept frozen in a -20^°^C freezer and thawed before use. A peristaltic pump (Masterflex model No. 7553–80, Cole-Parmer Instrument Co., IL., USA) was used in feeding and sampling operations to avoid introducing air into the digester. Both influent and effluent samples were analyzed for pH, TS and chemical oxygen demand (COD, colorimetric method) according to Standard Methods (APHA et al., 1998). Also, biogas production was recorded using the wet test gas meter (W-NK-0.5, Shinagawa Co., Tokyo, Japan), and the methane composition was determined using a gas chromatograph (GC-8700T, China Chromatography Co., Taiwan) equipped with a thermal conductivity detector (GC-TCD) and a Porapak Q column, with helium as the carrier gas. The gas sample was analyzed and compared with a gas standard. Liquid samples were kept frozen until analysis was conducted. The effluent for each test was frozen for later use.

### DNA extraction

Bacterial DNA was extracted from each collected sample using the PowerSoil^®^ DNA Isolation Kit (Mo Bio Laboratories, USA), according to the manufacturer’s instructions. Concentrations of double stranded DNA in the extracts were determined by the Quant-iT dsDNA HS assay kit and the Qubit fluorometer (Invitrogen, Life Technologies, Carlsbad, CA., USA). DNA was quantified with a Nanodrop-1000 Spectrophotometer (Thermo Scientific, Wilmington, DE, USA). All procedures were performed in laminar flow cabinet to avoid contamination.

### PCR amplification of 16S rRNA and sequencing

To increase primer coverage of sequences available in the Ribosomal Database Project (RDP) database, the primers used for DNA amplification were a modified 341F (CCTAYGGGRBGCASCAG) and a modified 806R (GGACTACNNGGGTATCTAAT) [37] fused with Illumina overhanging adapters, which amplified a DNA fragment of about 533 bp length flanking the V3 and V4 regions of the 16S rRNA gene [38]. The PCR amplification (30 μL final volume) was performed using 2X Phusion Flash High-Fidelity PCR Master Mix (Finnzymes Oy, Finland), and the incubation conditions were as follows: 98°C for 3 min, followed by 25 cycles of 98°C for 30 s, 56°C for 30 s, and 72°C for 30 s with a final extension of 72°C for 5 min. All PCR products were confirmed by 2% agarose gel electrophoresis and purified using NucleoSpin^®^ Gel and PCR Clean-up (Macherey-Nagel GmbH & Co. KG, Düren, Germany). DNA concentrations and quality of the cleaned PCR products were determined using the Nanodrop-1000 Spectrophotometer (Thermo Scientific, Wilmington, DE, USA).

The purified amplicons were further processed according to the Illumina standard protocol of 16S rRNA sequencing library preparation, and sequenced by the MiSeq platform with the reagent kit v3.

### Sequence processing

All paired-end sequences in FASTQ format were processed with FLASH software [39], MOTHUR v.1.36.1 [40, 41], and the UPARSE OTU (operational taxonomic units) analysis pipeline [42]. At first, these paired-end reads were merged using FLASH software [39] with standard parameters, with the exception of the maximum overlap parameter, which was set at 150. The successive filtering step for merged reads was performed by MOTHUR software [40]. With trim.seqs command, primer sequences were removed and all sequences less than 375 bases, with a quality score smaller than 30 or with homopolymers longer than eight nucleotides were discarded. These trimmed sequences were simplified by using the unique.seqs command to generate a unique set of sequences, and were clustered with a 97% similarity cutoff using UPARSE [42], where chimeric sequences were removed simultaneously with OTU-picking. The taxonomic assignment of the OTUs was achieved by the classify.seqs script using the trainset14_032015.rdp in MOTHUR [40] which used the RDP classifier with a confidence score threshold of 80%. The final step was to remove OTUs derived from Chloroplast, Mitochondria, Eukaryota, or unknown kingdom, and to perform the 16S rRNA gene copy number (GCN) adjustment of taxon abundance [43], and to generate an OTU table.

### Statistical analysis and classification of microbial members

The classification of microbial members was based on the OTU abundance. If OTUs contained more than 1% of the total number of sequences, they were denoted as high-abundance OTUs. If the abundance of OTUs in a time-series sample ranged from 0.1% to 1%, they were classified as low-abundance non-rare OTUs, and the remainder as rare OTUs.

There are *i* = 1,…,*N* OTUs and *k* = 1,…,*t* time-series samples in a microbial abundance table. The mean value of relative abundance, *x*_*ik*_, for an OTU *i* is defined below.

$${MEAN}_{i}=\frac{\sum_{k=1}^{t}\left(x_{ik}\right)}{N(x_{ik}>0)}$$ (1)

Differential OTUs between mesophilic and thermophilic anaerobic reactors were selected by the following steps. First, for each OTU among all time-series samples, the fold changes and relative abundance from two reactors were calculated. If there were missing values, the corresponding fold changes were ignored (*Eq (2)*). When the abundance of the mesophilic condition was used as a reference, the differentially abundant OTUs at thermophilic temperature could be identified. On the contrary, setting the thermophilic condition as the reference allows identification of the differentially abundant OTUs at mesophilic temperature.

$${FOLD}_{i}^{k}=\frac{x_{ik}^{treatment}}{x_{ik}^{reference}}\;if\;x_{ik}=0,\;then\;{FOLD}_{i}^{k}=0$$ (2)

Next, we determined the differential status of a fold change, *FOLD*_*i*_^*k*^, in each time-series sample (*Eq (3)*). If the fold change was larger than the threshold, setting 2 in this study, it was considered to be in a differential status.

$${INDEX}_{i}^{k}=\left\{ \begin{array}{cc} 1 & if\;{FOLD}_{i}^{k}\geq {FOLD}_{threshold}\\ 0 & if\;{FOLD}_{i}^{k}<{FOLD}_{threshold} \end{array}\right.$$ (3)

Finally, *Eq (4)* was to identify whether an OTU *i* belonged to the differentially abundant group. There were a total of *t* differential statuses among time-series samples for each OTU which could be divided into two time-series stages, including an early stage where *k* = 1,…,*t*_*cut*_ and a late stage where *k* = *t*_*cut+1*_,…,*t*. In this study, there were ten time-series samples in each anaerobic reactor and *t*_*cut*_ was set to 7. If there were over 4 differential fold changes in the early stage and over 2 in the late stage, this OTU was defined as a differentially abundant OTU under the treatment condition (see *Eq (2)*). This kind of settings is done in order to avoid identifying differential OTUs which only have differential abundances in the early stage.

$$\begin{array}{c} {OTU}_{i}^{diff}=(\sum_{k=1}^{t_{cut}}{INDEX}_{i}^{k}+\sum_{k=t_{cut+1}}^{t}{INDEX}_{i}^{k})\geq 6\\ \;if\;\sum_{k=1}^{t_{cut}}{INDEX}_{i}^{k}\geq 4\;and\;\sum_{k=t_{cut+1}}^{t}{INDEX}_{i}^{k}\geq 2 \end{array}$$ (4)

Furthermore, rarefaction curves or Shannon indices were constructed using the command rarefaction.single or collect.single in MOTHUR [40] to compare the microbial richness or diversity between the mesophilic or thermophilic processes.

### Inference for microbial interaction network

An OTU table is exhaustively divided into two groups according to whether an OTU is associated with a biogas related pathway (BRP) or not (NBRP). In the BRP or NBRP groups, there were *N*_*BRP*_ or *N*_*NBRP*_ microbial families used to generate a high confidence BRP interaction network by using a discrete-time LV model [44] coupled with a partial least square regression [45]. The high confidence BRP interaction network is a combination of two kinds of interaction networks. The first one is the raw BRP interaction network derived from the BRP group which constitutes an interaction network with (*N*_*BRP*_) × (*N*_*BRP*_−1) interactions where $M_{ij}^{BRP}$ represents a repressive or active effect of OTU *j* on *i*. The second one is a group of mixed interaction networks from both the BRP and NBRP group over *N*_*sim*_ simulation runs. For each simulation, $N_{\tilde{NBRP}}$ OTUs are randomly selected from the NBRP group and combined with *N*_*BRP*_ OTUs from the BRP group to generate an interaction network where $M_{ij,sim}^{BRP+\tilde{NBRP}}$ is the interactive relationship. Over the *N*_*sim*_ simulated interaction networks, an interactive relationship is identified if one of the following conditions is fulfilled when *P* is defined by *Eq (5)*.

$$P=\frac{\sum_{sim=1}^{N_{sim}}\left(M_{ij,sim}^{BRP+\tilde{NBRP}}>0\right)}{N_{sim}}$$ (5)

### Condition I

If *P* ≥ *P*_*threshold*_ and $M_{ij}^{BRP}>0$, then $M_{ij}^{BRP}$ reveals an active confident interactive relationship of OTU *j* and *i*.

### Condition II

If *P* ≥ *P*_*threshold*_ and $M_{ij}^{BRP}\leq 0$, then $M_{ij}^{BRP}$ reveals a non-confident interactive relationship.

### Condition III

If *P* ≤ 1 − *P*_*threshold*_ and $M_{ij}^{BRP}\geq 0$, then $M_{ij}^{BRP}$ reveals a non-confident interactive relationship.

### Condition IV

If *P* ≤ 1 − *P*_*threshold*_ and $M_{ij}^{BRP}<0$, then $M_{ij}^{BRP}$ reveals a confident repressive interactive relationship of OTU *j* and *i*.

In our study, 173 microbial members were identified at the family level, comprised of 36 BRP and 137 NBRP families. There were a total of 1260 interactions in the raw BRP interaction network ($M_{ij}^{BRP}$). Then *N*_*sim*_ = 1000 mixed interaction networks were generated by 1000 simulation runs where $N_{\tilde{NBRP}}$ = 120 NBRP families were randomly chosen from the NBRP group and cooperated with *N*_*BRP*_ = 36 BRP families to infer interactive relations ${(M}_{ij,sim}^{BRP+\tilde{NBRP}})$ for each simulation run. Over the 1000 simulated interactive networks, an interactive relation coherent in *P*_*threshold*_ = 95% of simulated outcomes and consistent with the raw BRP interactive relation ${(M}_{ij}^{BRP})$ was defined as high confidence. Finally, there were 689 and 671 high confidence interactions in the mesophilic or thermophilic anaerobic digesters, respectively.

### Topological inference for microbial interaction network

The topological analysis of an interaction network was conducted by three measurements using Gephi software [46]. Measurements included in-degree centrality, betweenness centrality and eigenvector centrality. In a topological interaction network, a node represents an OTU *i* and an edge indicates the interactive relation between two OTUs. In-degree centrality quantifies the number of nodes that an OTU receives. Larger the values, indicate that the OTU is influenced by more OTUs or interactions. In-degree centrality is displayed by node color, the darker (or lighter) red indicates that the OTU has more (or less) in-degree relationships. Betweenness centrality measures the number of times an OTU functions as a bridge along the shortest path between two other OTUs. Therefore an OTU has a strong betweenness centrality (bigger node size) if it bridges on many shortest paths. Eigenvector centrality is a measurement to identify the importance of an OTU in a network based on its connections. An important OTU is always linked by other important OTUs and situated at the core of the network.

### The biological evaluation of inferred microbial interactions

In the tool, MetaMIS [27], the microbial interactions were evaluated using two metabolic indices including complementarity and competition index. The metabolic complementarity index is defined as the fraction of metabolites in one species’ nutritional profile but not in the nutritional profile of another one [47] in which a nutritional profile is retrieved from Kyoto Encyclopedia of Genes and Genomes (KEGG) metabolic pathways [48]. The index ranging from 0 to 1 measures the trophic dependence between two microbes. Thus, an index of 1 means that all of the nutrients required by one microbe can be synthesized by another microbe from metabolic precursors. The metabolic competition index is defined as the fraction of metabolites in a species’ nutritional profile that are also included in another species’ nutritional profile. This index ranges from 0 to 1 as a measure of the trophic competition between two microbes; thus an index of 1 means that all of the nutrients required by two microbes are the same. According to genome-scale metabolic network models, 23,562 metabolic complementarity or competition indices for pairs of microbial species [47] were transformed to 19,182 microbial trophic relations at the family level. For each transformation, metabolic indices from the same family were averaged.

## Results

### Biogas production in mesophilic or thermophilic AnD

**Fig 1:**
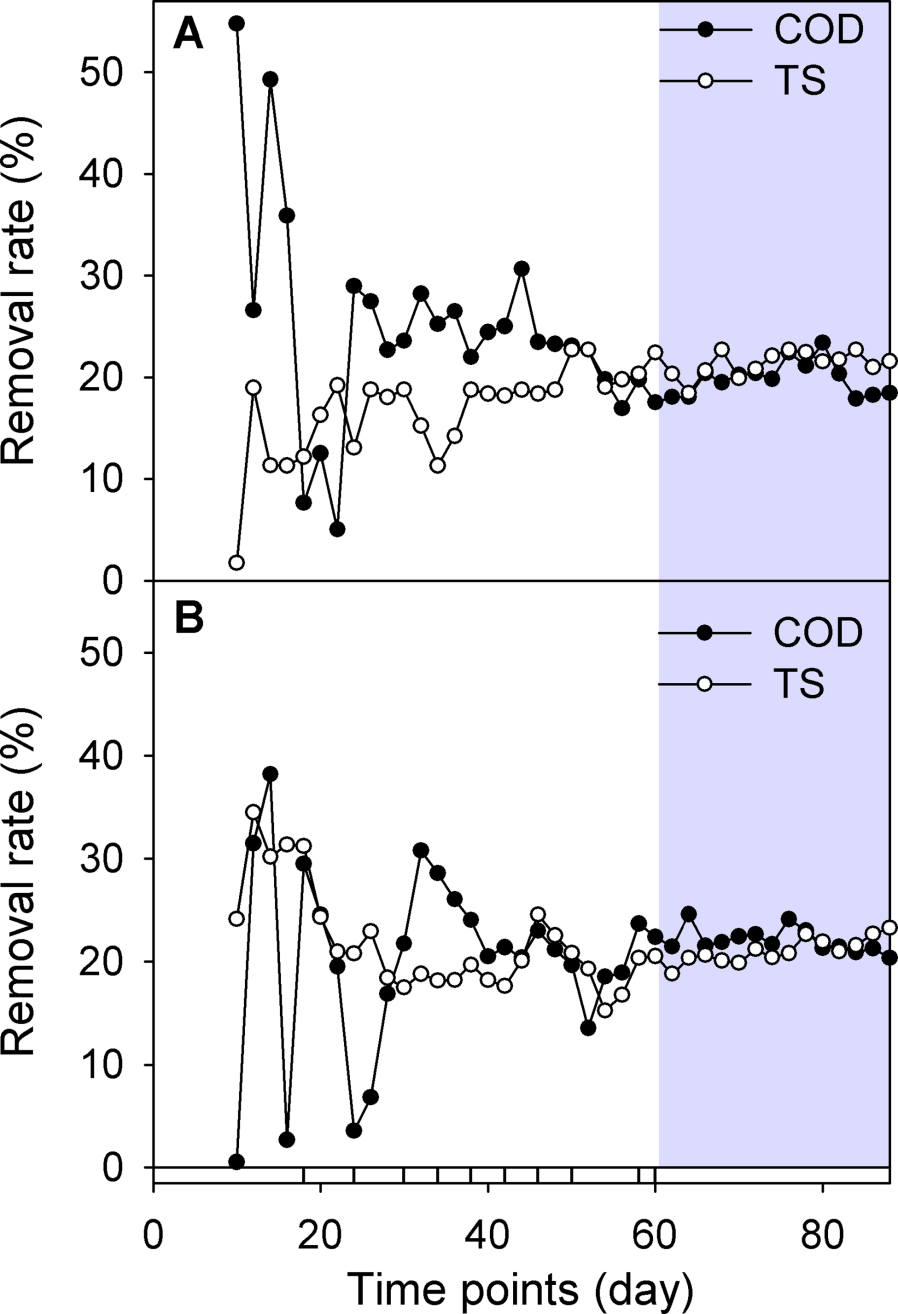
The fluctuation of COD and TS over time. Ten time points were selected according to the changing rate of COD and TS in the MAnD (A) or TAnD (B) digesters as labeled in the bottom of the diagram. The gray region indicates the stable duration of COD/TS removal rates (i.e., RCOD or RTS).

**Fig 2:**
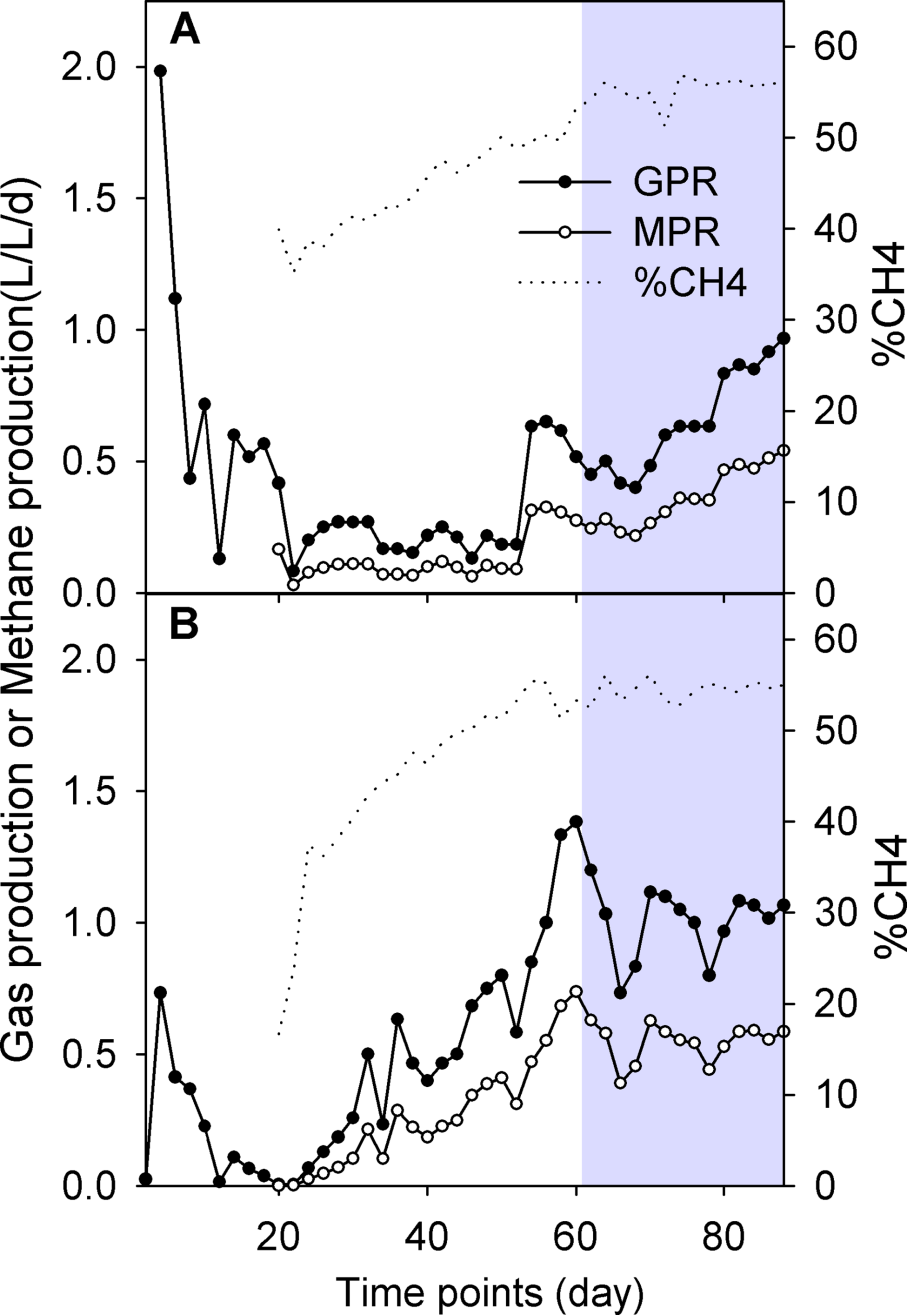
Biogas production in the anaerobic reactors. MAnD (A) or TAnD (B) digesters. (A) The MAnD digester had lower efficiency of gas (-●-) or methane (-○-) production than the TAnD one (B). The percentage of methane (CH_4_%) in biogas was similar in the two reactors.

During the 90 days of operation of the AnD processes, the removal efficiency of COD and TS in the MAnD or TAnD anaerobic reactors became more stable after the sixtieth day (Gray region in Fig 1). These facts indicate a steady state of organic carbon mineralization into biogas under mesophilic or thermophilic conditions (Fig 1 and S1 Table). As shown in Fig 2, biogas and methane from thermophilic conditions (Fig 2B) were produced at a higher level than that obtained from the mesophilic digester (Fig 2A) at any period (S1 Table). Methane yields accounted for a similar fraction of the total biogas collected under mesophilic or thermophilic conditions in all time periods (Fig 2), with a maximum methane content of 55% at the end of each steady-state period.

### Summary of sequence data and archaeal percentage

We used Illumina-based 16S rRNA sequencing to examine the complex microbial communities found in the AnD digesters. In total, 20 effluent samples at ten time points were collected from MAnD or TAnD reactors before the steady-state period (Fig 1). This obtained approximately 3 million paired-end reads (~900 megabases of 16S rRNA amplicon sequence). All sequenced samples which had been deposited in the NCBI Short Read Archive under BioProject PRJNA350355 (SRR4450465- SRR4450484) showed similar levels of sequence quality. After pre-processing steps of quality control, paired-end merging, primer trimming, and taxonomic assignment, about 75%, i.e. 1,124,760 quality-checked reads, from the total raw reads were retained for later analysis (Table 1).

**Table 1:** The number of pyrosequencing reads after preprocessing. The percentage of total sequence reads is shown in brackets.

	37^°^C	55^°^C
**Total Reads**	66019±5089 (100%)	83293±6987 (100%)
**Merged Reads**	59658±4626 (90.4%)	75131±6314 (90.2%)
**Trimmed Reads**	52474±4081 (79.5%)	66171±5608 (79.4%)
**Final reads**	50499±3952 (76.5%)	62157±5472 (74.6%)

Between 86.9% and 91.7% of all the sequences were assigned to the domain bacteria in the mesophilic samples, while between 92.1% and 97.8% of sequences were assigned at thermophilic temperature (Table 2). The percentage of archaeal sequences (8.3–13.1%) at mesophilic temperature was higher than in the thermophilic conditions (2.2–7.9%), especially after performing GCN correction (Table 2). All of the archaeal or bacterial sequences were classified to family-equivalent OTUs recognized in the RDP database. In total, 173 major families were identified after discarding 5 or 19 OTUs uniquely in the thermophilic or mesophilic conditions respectively.

**Table 2:** The archaeal percentage (%) with or without GCN correction, denoting GCN or NGCN, respectively.

	T_18_	T_24_	T_30_	T_34_	T_38_	T_42_	T_46_	T_50_	T_58_	T_60_
**37**^**°**^**C**_NGCN_	8.4	10.7	13.1	9.1	8.3	7.5	8.4	10.7	10.7	12.7
**55**^**°**^**C**_NGCN_	6.4	5.5	2.2	4.0	3.5	2.4	3.0	5.0	7.9	6.8
**37**^**°**^**C**_GCN_	13.0	16.3	19.6	14.0	12.8	11.6	12.9	16.3	16.2	19.1
**55**^**°**^**C**_GCN_	10.1	8.7	3.5	6.3	5.6	3.9	4.8	7.8	12.2	10.6

### Comparison of microbial community structure

#### Rarefaction curves and microbial diversity

Rarefaction curves of observed OTU richness revealed the saturation of the number of OTUs from the asymptotic nature of randomly sampled reads (S1 Fig), indicating that the number of sequences per time-series sample was high enough to cover the microbial diversity in each sample. The estimated number of OTUs was apparently lower under thermophilic conditions although the sequencing depth was higher. In spite of the fact that microbial richness was influenced by temperature (S1 Fig), a lower Shannon diversity index was observed under thermophilic anaerobic environments in all experimental periods (S2 Fig).

#### Biogas-related microorganisms

Microorganisms from the bacterial or archaeal domains execute several interdependent, successional, and complex biological interactions to influence the efficacy of methanogenesis. As the best characterized methane producers in AnD, different methanogenic families were dominant at different temperatures; *Methanotrichaceae*, *Methanocorpusculaceae*, *Methanoregulaceae* and *Methanomassiliicoccaceae* were abundant at the mesophilic temperature. However, under thermophilic conditions *Methanobacteriaceae* and *Methanomicrobiaceae* became more prominent (Table 3). Methanogenic anaerobes often cooperate with bacterial consortia which play key roles to balance various biological conversion processes (i.e. BRP) in AnD, for example hydrolysis, acidogenesis, acetogenesis, methanogenesis and desulfurization, in order to avoid the accumulation of inhibitory end products. Supported by the literature [49–53], we identified 36 microbial families which participated in BRP. The remainders (137 families) were placed into the non-biological conversion process (i.e. NBRP) group.

**Table 3:** The relative abundance of microorganisms in the BRP group. All values are shown as % of total sequence in each sample, except of the rightest column.

Taxonomy in family level	Seed^a^	Manure^a^	37^°^C ^a^	55^°^C^a^	Diff^b^	N_genus_
p1: *Porphyromonadaceae*	0.32^L^	23.60^H^	3.11^H^	2.56^H^	-	8
p1: *Spirochaetaceae*	0.49^L^	2.28^H^	2.06^H^	0.90^L^	DN	2
p1: *Rikenellaceae*	-	<0.01^R^	0.01^R^	0.06^R^	-	3
p1: *Bacteroidaceae*	-	0.92^L^	0.08^R^	0.06^R^	-	2
p2: *Flavobacteriaceae*	0.02^R^	0.29^L^	0.42^L^	0.63^L^	-	9
p2: *Enterococcaceae*	-	0.26^L^	0.02^R^	<0.01^R^	DN	1
p2: *Veillonellaceae*	0.02^R^	-	<0.01^R^	0.01^R^	-	1
p2: *Aerococcaceae*	<0.01^R^	0.12^L^	<0.01^R^	-	-	2
p3: *Erysipelotrichaceae*	0.09^R^	2.59^H^	2.13^H^	3.47^H^	-	5
p3: *Peptostreptococcaceae*	0.23^L^	9.51^H^	1.84^H^	3.19^H^	-	5
p3: *Syntrophaceae*	5.79^H^	0.04^R^	0.69^L^	0.16^L^	-	3
p3: *Acidaminococcaceae*	<0.01^R^	<0.01^R^	0.14^L^	0.26^L^	-	3
p3: *Desulfovibrionaceae*	0.02^R^	<0.01^R^	0.02^R^	0.01^R^	-	3
p4: *Methanotrichaceae*	20.42^H^	0.03^R^	6.46^H^	1.45^H^	DN	1
p4: *Methanobacteriaceae*	0.40^L^	0.04^R^	0.68^L^	2.14^H^	UP	3
p4: *Methanosarcinaceae*	0.24^L^	0.06^R^	1.47^H^	1.90^H^	-	5
p4: *Corynebacteriaceae*	0.02^R^	8.71^H^	1.05^H^	1.29^H^	-	1
p4: *Methanomicrobiaceae*	0.02^R^	-	0.12^L^	1.54^H^	UP	2
p4: *Methanocorpusculaceae*	0.12^L^	0.11^L^	2.46^H^	0.12^L^	DN	1
p4: *Pseudomonadaceae*	0.04^R^	17.55^H^	0.28^L^	0.84^L^	UP	3
p4: *Methanoregulaceae*	21.61^H^	0.02^R^	0.83^L^	0.25^L^	DN	3
p4: *Methanospirillaceae*	1.89^H^	0.11^L^	0.25^L^	0.10^L^	-	1
p4: *Enterobacteriaceae*	<0.01^R^	1.55^H^	0.11^L^	0.28^L^	UP	2
p4: *Planococcaceae*	-	-	0.01^R^	0.21^L^	UP	6
p4: *Coriobacteriaceae*	<0.01^R^	0.35^L^	0.15^L^	0.14^L^	-	6
p4: *Methanomassiliicoccaceae*	1.13^H^	-	0.14^L^	0.06^R^	DN	1
p1,2: *Clostridiaceae_1*	0.61^L^	11.65^H^	1.77^H^	2.96^H^	-	3
p1,2: *Clostridiaceae_2*	-	-	<0.01^R^	<0.01^R^	-	1
p2,3: *Lactobacillaceae*	<0.01^R^	0.53^L^	<0.01^R^	<0.01^R^	-	1
p1-4: *Ruminococcaceae*	1.21^H^	1.79^H^	13.51^H^	10.33^H^	-	15
p1-4:*Prevotellaceae*	-	0.01^R^	0.39^L^	1.03^H^	-	2
p1-4:*Lachnospiraceae*	0.04^R^	2.23^H^	0.34^L^	1.39^H^	UP	5
p1-4:*Synergistaceae*	2.46^H^	0.02^R^	1.59^H^	0.54 ^L^	DN	6
deS: *Syntrophobacteraceae*	0.43^L^	-	0.23^L^	0.12 ^L^	-	2
deS: *Desulfobulbaceae*	0.02^R^	0.01^R^	0.03^R^	0.02 ^R^	-	1
deS: *Desulfobacteraceae*	0.03^R^	-	<0.01^R^	<0.01 ^R^	-	1
**Eight methanogens**	45.84	0.36	12.39	7.55		
**36 BRP families**	57.67	84.38	42.39	38.06		

^a^ H: high abundance; L: low abundance; R: rare abundance (details see materials and methods)

^b^ Diff indicates the differentially abundant OTUs at thermophilic or mesophilic temperature.

-: Microbial abundance is similar between the two anaerobic digesters.

DN (or UP): Microbial abundance is lower (or higher) at thermophilic temperature.

**Fig 3:**
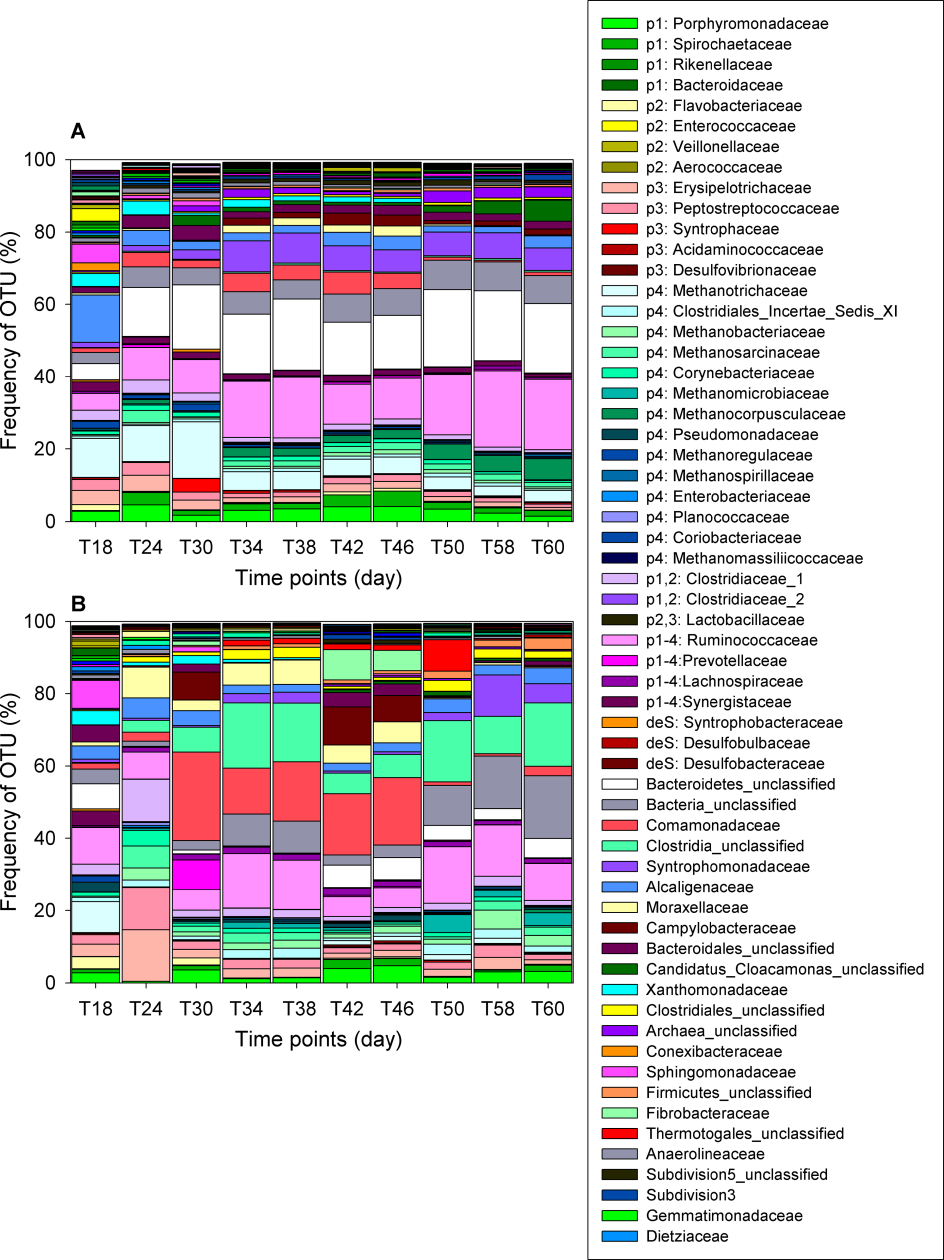
Different microbial compositions at MAnD or TAnD digesters. Microbial communities shown at the family level under mesophilic (A) or thermophilic (B) conditions. 36 BRP microbial families related to five biogas generation pathways were denoted as p1 for hydrolysis, p2 for acidogenesis, p3 for acetogenesis, p4 for methanogenesis, and deS for desulfurization.

Representatives of 36 bacterial or archaeal families involved in BRP were detected (Table 3 and Fig 3). We examined another two microbial communities from the seeds of digested manure sludge and influent pig manure as controls to trace microbial temporal changes. The accumulated relative abundance of methanogens revealed that seed sludge was the major source of methanogens (45.84%) for those from MAnD (12.39%) or TAnD (7.55%) digesters (Table 3). However, influent substrates from swine manure constituted a bacterial repository and provided 84.02% of its community during the process of hydrolysis, acidogenesis, acetogenesis and desulfurization (Table 3).

#### Temperature-dependent changes in microbial community composition

Of the 173 OTUs identified at the family level, we identified 13 differentially abundant OTUs that were involved in the BRP group (Table 3) and 15 from the NBRP group (S2 Table). In the TAnD reactor, there were six differentially abundant families that participate in methanogenesis; *Methanobacteriaceae*, *Methanomicrobiaceae*, and *Lachnospiraceae* were the most common of the differentially abundant families, conveying 2.14%, 1.54%, and 1.39% of total reads respectively (Table 3). *Pseudomonadaceae*, *Enterobacteriaceae* or *Planococcaceae*, as low-abundance families, accounted for only 0.84%, 0.28% or 0.21% of the total prokaryotic communities, respectively. Interestingly, *Planococcaceae* could not be detected in the seed sludge or swine manure but was detectable, albeit at very low relative abundance under thermophilic conditions. In contrast, in the MAnD process, four methanogens, *Methanotrichaceae* (6.46%), *Methanocorpusculaceae* (2.46%), *Methanoregulaceae* (0.83%) and *Methanomassiliicoccaceae* (0.14%), were highly abundant families compared with thermophilic conditions. The major source of *Methanotrichaceae*, *Methanoregulaceae* and *Methanomassiliicoccaceae* was from seed sludge because their relative abundance in seed sludge reached 20.42%, 21.61%, and 1.13% respectively. However, these high-abundance donor communities did not remain present at high levels during the start-up period of reactors (Fig 3). *Methanocorpusculaceae* which was equally present at relatively low abundance in seed sludge (0.12%) or swine manure (0.11%) remained at a constant level at the higher temperature, and reached 2.46% at the mesophilic temperature. Other than methanogens, two differentially abundant bacterial families were identified; *Spirochaetaceae* (2.06%) and *Enterococcaceae* (0.02%) which function in hydrolysis and acidogenesis respectively to enhance the AnD degradation of organic matter (Table 3). Specifically, extracts from mesophilic or thermophilic conditions had respective differentially abundant families, *Lachnospiraceae* or *Synergistaceae*, which have a wide spectrum of mechanisms that may affect the overall phases of the BRP processes. There was no differential family identified which was involved in the process of desulfurization (Table 3). The differentially abundant taxa that do not participate in the BRP process are listed in S2 Table. The detailed metabolic pathway [48] that microbes of the BRP group was shown in S3 Table.

### Different microbial interaction networks

**Fig 4:**
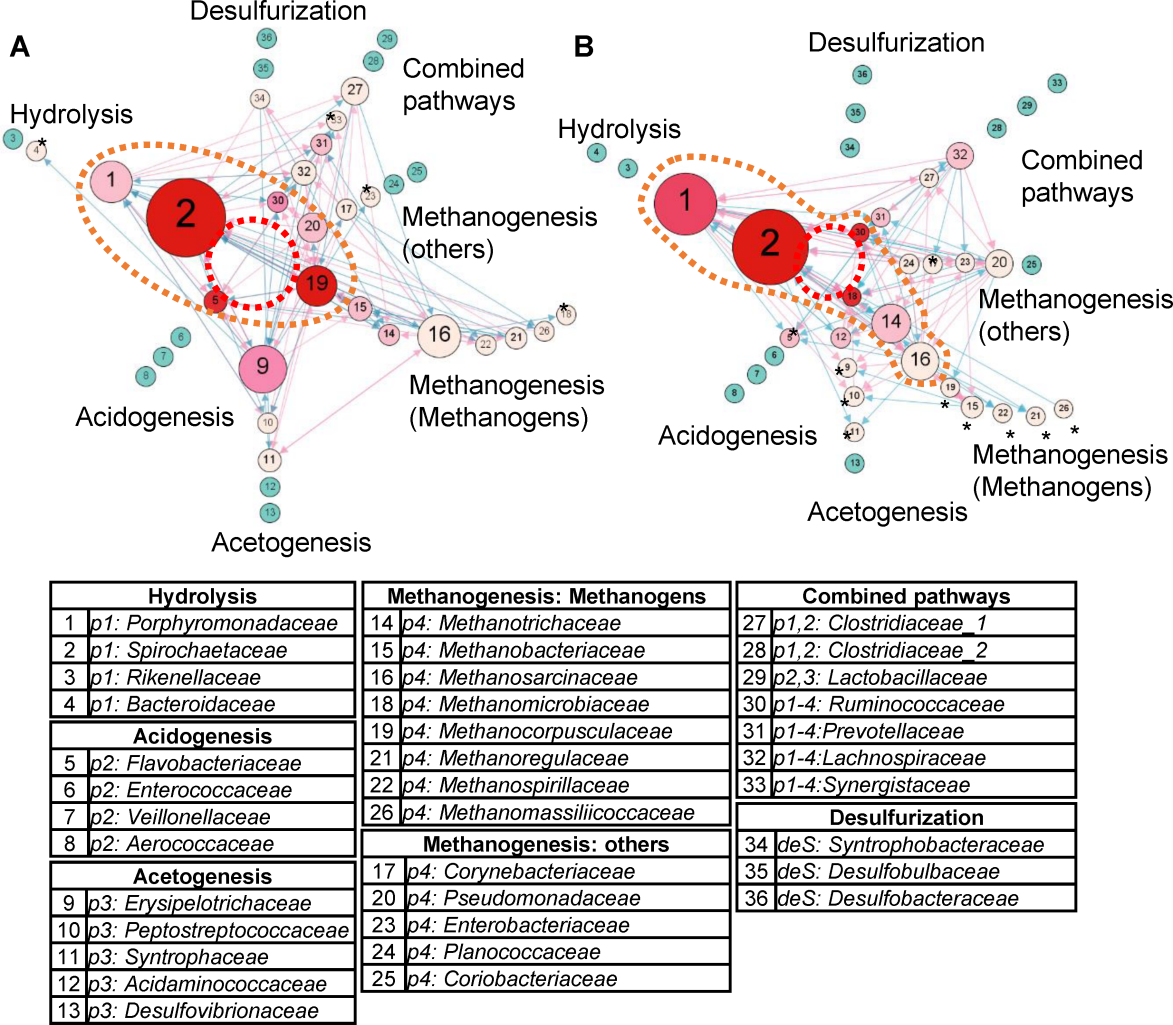
Microbes with different topological niches under mesophilic or thermophilic conditions. The interaction networks identified at mesophilic (A) or thermophilic (B) temperatures. Nodes represent microbial families. The size of the nodes corresponds to their betweenness centralities. A larger node indicates higher betweenness centrality which has a large influence on the transfer of information through the network. The color of the nodes conveys the number of in-degree interactions, describing how many OTUs influence this node. The node shown in dark red connects the maximum in-degree nodes, i.e., 10. The node closest to the center of the network has the highest level of eigenvector centrality, a measure of the influence of a node in a network. Three nodes linked by a red dotted circle indicate the top three most influential OTUs. The top six are denoted inside an orange dotted circle. The pink arrow indicates an activate relationship, and the blue arrow indicates a repressive event. Nodes marked with an asterisk (*) have a very low level of out-degree interactions.

In this study, temperature was a critical factor influencing the microbial abundance and composition. At mesophilic temperature, the microbial community had higher diversity and more differentially abundant families involved in BRP processes. However, under thermophilic conditions, the differentially abundant taxa were most concentrated in the process of methanogenesis. These observed differences in microbial communities might be controlled by the intrinsic interactions between microorganisms. To visualize the structure change, totally 173 families were used to infer the microbial interactions, the interaction networks with the 100 strongest interactive strengths from the MAnD or TAnD reactors were sketched (Fig 4, only the 36 BRP group was shown). Using eigenvector centrality, *Flavobacteriaceae*, *Methanocorpusculaceae* and *Spirochaetaceae* connected by central OTUs were the most influential in the mesophilic interaction network (dotted red circle in Fig 4A). Under thermophilic conditions, the most influential OTUs were *Spirochaetaceae*, *Ruminococcaceae*, and *Methanomicrobiaceae* (dotted red circle in Fig 4B). *Spirochaetaceae* played a critical role and was common between the two reactors, where the color of dark red indicated it was regulated by more microbial members and the large node size, conveying high betweenness centrality, indicates that *Spirochaetaceae* was always connected to other microbial members with the shortest paths. The other four members which were distinct between mesophilic and thermophilic processes, *Flavobacteriaceae*, *Methanocorpusculaceae*, *Ruminococcaceae* and *Methanomicrobiaceaei*, had concordant topological properties such as a smaller node size with a dark red color: these were regulated by a variety of microorganisms but rarely influenced others. Within the top six microbial families with high eigenvector centrality (dotted orange circle in Fig 4), the leading methanogens were very distinct between the two reactors, *Methanocorpusculaceae* was critical at mesophilic temperature, and *Methanomicrobiaceae*, *Methanotrichaceae* and *Methanosarcinaceae* were important in the thermophilic reactors. Furthermore, more OTUs denoted with stars were observed at high temperature. These nodes with stars were unlikely to influence other members owing to the low level of out-degree interactions.

In two reactors, there were several concordant observations based on the topological structure. *Porphyromonadaceae* and *Spirochaetaceae* were highly regulated by the microbial community and also gave feedback to the microbial society. However, *Methanosarcinaceae* mainly influenced the community but was rarely influenced by others. In contrast, the major role of *Ruminococcaceae* was to receive various stimuli from other members especially in thermophilic conditions. Regardless of abundance measurements, *Methanospirillaceae*, *Methanoregulaceae* and *Methanomassiliicoccaceae* contributed consistently with the least impact in both interaction networks.

Based on relative abundance, there were two differentially abundant methanogens and four bacteria identified in thermophilic conditions (Table 3). After transforming the abundance profile to an interaction network, a variety of influential bacteria or methanogens were identified in both reactors. For example, *Methanosarcinaceae* had non-differential relative abundance in the two reactors but existed in critical topological situations. Consequently, the network-based approach provides a novel view to decipher changes in microbial communities between the two different temperatures.

### Metabolic support for the microbial interactions

**Fig 5:**
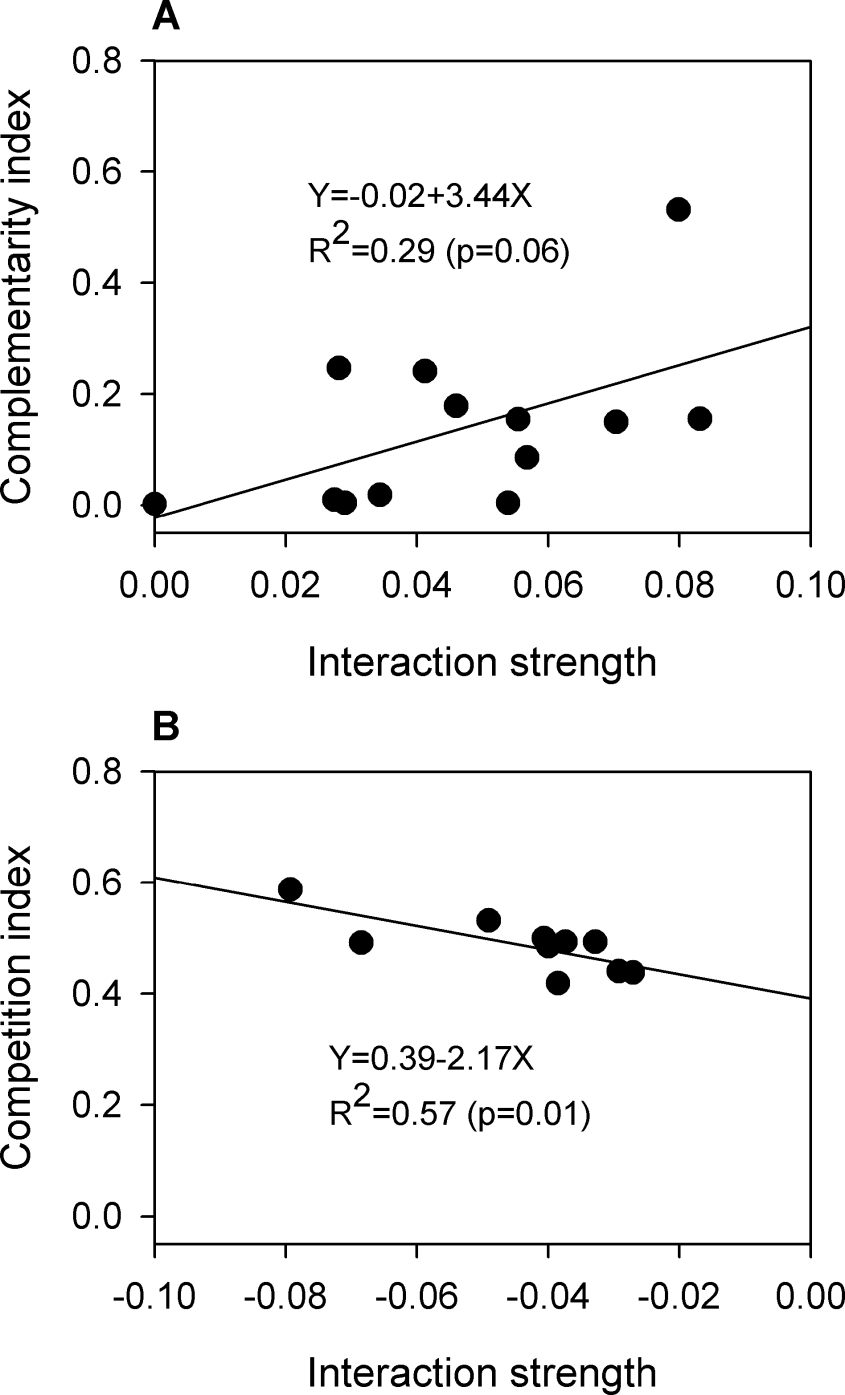
The association between metabolic trophic relations and microbial interactions. (A) Metabolic complementarity index had a borderline association with positive interactive strengths. (B) Metabolic competition index showed a statistically significantly association with negative interactive strengths.

Organizing the trophic relations, metabolic complementarity or competition index, into mesophilic microbial interactions in MetaMIS [27] was manipulated to evaluate the underlying biological connections. For the 100 strongest interactions from the MAnD reactors (Fig 4), only 23 interaction pairs (S4 Table) can be mapped with trophic information due to the database limitation. Nevertheless, the inferred strengths of positive interactions revealed a borderline association (R^2^ = 0.29; p-value = 0.06) with their metabolic trophic dependences (Fig 5A). Similarly, changes of inferred negative interactive strengths are statistically significantly associated (R^2^ = 0.57; p-value = 0.01) with changes of metabolic competition indices (Fig 5B). The results suggested that the inferred microbial interactions were supported by the measurement using their metabolic properties.

## Discussion

The aim of our study was to use deep sequencing technology to investigate the interactions in microbial communities in mesophilic or thermophilic reactors generating biogas from hog waste. Analysis of bacterial and archaeal communities in mesophilic or thermophilic reactors displayed clear differences as regards methane yields, microbial richness, Shannon diversity, archaeal percentage, differential abundance of families, and interaction networks. From the surrounding literature on the microbiome from MAnD or TAnD [10, 54, 55], it appears that, in general, thermophilic anaerobic digesters conveyed a higher level of methane yield, pH, COD or TS removal efficiency [54], and a lower level of species richness, biodiversity [54, 55] or archaeal percentage [10, 55], when compared with MAnD reactors. Despite the level of pH, COD or TS removal efficiency, our findings were in agreement with the results of these previous studies. The lower microbial richness and diversity under thermophilic conditions have been explained by the disinfection of pathogenic microbes and efficient biodegradation of animal manure [56]. The present study measured organic matter degradation through quantifying the reduction of COD or TS. The higher level of TS digestion at the thermophilic temperature implied the degradation of complex organic particulates and the accumulation of volatile fatty acids including formic acid, acetic acid, propionic acid, or butyric acid, etc., which may lower the pH of AnD reactors [57]. Here, we observed a very stable pH level which as a whole ranged from pH 7.1 to 7.2 not only in mesophilic but also in thermophilic digesters. Thus, we supposed that higher temperature did not increase the microbial population involved in hydrolysis or acidogenesis but might result in an early microbial shift towards acetogenesis or methanogenesis according to three current observations described in details below.

First, there were no differentially abundant families from the thermophilic conditions identified which are known to function in hydrolysis or acidogenesis. Second, the cumulative relative abundance of acetogenic bacteria at the thermophilic temperature reached 7.09% higher than that observed in mesophilic conditions (4.83%). Thirdly, differentially abundant families at the TAnD reactor gathered in the methanogenic pathway, which constituted a hydrogenotrophic methanogen of *Methanobacteriaceae*, a hydrogenotrophic/methylotrophic of *Methanomicrobiaceae*, and three low-abundance microorganisms, including *Enterobacteriaceae* affiliated with hydrogen-producing processes [58], *Planococcaceae* associated with a high concentration of carbon source [59] and *Pseudomonadaceae* which could reduce Fe(III) with hydrogen or acetate [60]. In contrast, under mesophilic conditions, the differentially abundant bacteria were broadly located in hydrolysis, acidogenesis, and acetogenesis and only methanogens had significantly higher relative abundance in methanogenesis. This can be explained by the high digestion efficiency of organic matter at higher temperatures and not necessarily an increase in the bacterial community responsible for hydrolysis or acidogenesis.

Acetogenesis is a general metabolic pathway shared by several phyla including *Spirochaetes*, *Firmicutes*, *Chloroflexi*, and *Deltaproteobacteria* [61]. Although the reactions that involve in the carbon flow of the homoacetogenesis are highly conserved in all acetogenic bacteria, it was shown that homoacetogens produce either the Rnf (multisubunit ferredoxin–NAD^+^ oxidoreductase) or the Ech (energy-converting hydrogenase) complex to couple with the Wood–Ljungdahl pathway to generate a transmembrane ion (Na^+^ or H^+^) gradient that drives ATP synthesis [62]. Wood–Ljungdahl pathway is an intermediate process that produces acetate from inorganic gases like CO_2_ and H_2_ [63]. According to the energy transport system, there are three types of homoacetogens:Rnf-containing (Na^+^-dependent), Rnf-containing (H^+^-dependent), and Ech-containing (H^+^-dependent). The question of whether homoacetogenesis is prevalent under mesophilic [62] or thermophilic condition [63] remains a debate. According to the network topology analysis, we found the hints to explain the potentially roles of acetogneic bacteria in MAnD or TAnD. For example, based on the differential topological niche of *Acidaminococcaceae*, the homoacetogenesis conducted by Rnf-containing and Na^+^-dependent acetogenic mechanism may be more influential at the thermophilic condition than that at mesophilic condition [64]. It was possible that acetogens could utilize Rnf-containing and Na^+^-dependent acetogenic mechanism to avoid the competition of hydrogen with hydrogenotrophic methanogens at thermophilic condition. Similarly, according to network topology, the functional role as acetogen played by *Erysipelotrichaceae* might be differential between MAnD and TAnD. The exact functional mechanism of *Erysipelotrichaceae* can be identified when the genome sequencing is completed. Since the speculations were brought up based on the theoretical inference of microbial interactions, a further investigation is required to confirm the role of acetogenic microbes at MAnD or TAnD.

Although differential analysis had revealed a distinct microbial structure between MAnD and TAnD reactors, modeling microbial abundance profiles by LV equations [46] and topological analysis shed light on the intertwined relationships between microorganisms. The family *Methanotrichaceae* and *Methanosarcinaceae* which control the majority of methane production are two well-known aceticlastic methanogens which are capable of converting acetic acid into methane. *Methanotrichaceae*, also known as *Methanosaetaceae*, takes acetic acid as the only substrate for methanogenesis with the formation of methane and carbon dioxide but *Methanosarcinaceae* possesses a complicated trophic system that can convert hydrogen, methanol, methyl sulfide, monomethylamine, dimethylamine, trimethylamine, acetic or pyruvic acid to methane [65]. Generally, at thermophilic temperature, the hydrogen producing process becomes prevalent and favored at low acetate concentrations, while aceticlastic methanogenesis is favored by high acetate concentrations [66, 67]. The lower level (1.45%) of *Methanotrichaceae* at higher temperatures implied that the lower concentration of acetate than observed in the mesophilic digesters is due to the syntrophic association between hydrogenotrophic methanogens, including *Methanomicrobiaceae* and *Methanobacteriaceae*, and acetate-oxidizing bacteria, often the genus *Clostridium* [68] which was in the family *Clostridiaceae_1* in the current study, or other hydrogen-producing bacteria, such as *Ruminococcaceae* and *Prevotellaceae* [69]. The topological structure of *Ruminococcaceae* and *Prevotellaceae* was located at the core of network measured by eigenvector centrality and showed very essential occupations, especially in the thermophilic digesters. By utilizing the measurement of eigenvector centrality, describing the kind of representatives in a network, *Methanomicrobiaceae* or *Methanocorpusculaceae* were the most influential methanogens at thermophilic or mesophilic temperatures respectively. The literature supports the role of *Methanomicrobiaceae*, which has been identified under mesophilic and thermophilic conditions [70], and *Methanocorpusculaceae*, which favors mesophilic temperature [71], and the two methanogens are driven by sharing the common substrate source including hydrogen, formic acid, isopropanol or ethanol, etc [72]. Interestingly, the three methanogens with relatively non-influential topology were controlled by a simple substrate source, such as *Methanoregulaceae* (hydrogen and formic acid), *Methanospirillaceae* (formic acid) and *Methanomassiliicoccaceae* (methanol). Furthermore, the disappearance of interactive relationships observed for *Syntrophobacteraceae* in thermophilic conditions may be explained by its mesophilic tendency [73].

In conclusion, based on analysis of differential abundance, microorganisms were more involved in methanogenesis at thermophilic temperatures but distributed on diverse pathways under mesophilic conditions. This observation was also made by topological analysis. The greater number of nodes (OTUs) with a low level of out-degree interactions in the thermophilic interaction network indicated that interactions were concentrated on a smaller group of organisms at thermophilic temperature and more dispersed at mesophilic temperature.

## Conclusions

Research on methane production in AnD has been underway for decades, however, the microbial interactions in different AnD conditions have not yet been explored in any detail. According to previous observations in this study, we know that increasing temperature will change the microbial composition and decrease microbial diversity. Our development of topological interaction networks represents an early attempt to gain a systematic understanding of these microbial interactions and reveals more efficient topological relationships at thermophilic temperature. These predicted microbial interactions were not only biologically informative but also metabolically supported. The interaction network described here could serve as the basis for in-depth studies of microbial interactions, identification of the kind of biological mechanisms that drive these intertwined networks, and how microbes interact to raise methane yields. Nevertheless, more biological experiments are required to validate the inferences of microbial interactions to fill in the gaps between realistic and estimated phenomena. The network approach has been applied in gene expression or metabolic research, to successfully uncover gene-gene interactions [74] or metabolic networks [75, 76]. Our group pioneered the method to generate microbial interaction networks in order to study anaerobic digesters. We have provided several new insights to provide a foundation for further studies in this area. Such approach has many applications and potential in other research fields.

## Supporting information

S1 Fig Assessment of OTU coverage by rarefaction analysis. Individual rarefaction curves for each time-series sample taken from the MAnD (A) or TAnD (B) digesters.
(TIF)

S2 Fig Microbial community divergence in mesophilic and thermophilic temperatures. Diversity of microbial communities was represented as white or black circles for mesophilic (○) or thermophilic (●) conditions. The larger Shannon index represents a higher level of microbial diversity.
(TIF)

S1 Table Indicators of anaerobic digestion before or after day 60. (PDF)

S2 Table Differentially abundant families from each NBRP group under mesophilic or thermophilic conditions. (PDF)

S3 Table Metabolic pathways that 36 BRP families participated in. (XLSX)

S4 Table Interaction strength, complementarity and competition index under the mesophilic condition. (PDF)
