# Supplementary figures and images for: Inferring microbial interactions in thermophilic and mesophilic anaerobic digestion of hog waste
Source: PLoS One. 2017 Jul 21;12(7):e0181395. doi: 10.1371/journal.pone.0181395 (PMC5521784; doi:10.1371/journal.pone.0181395)

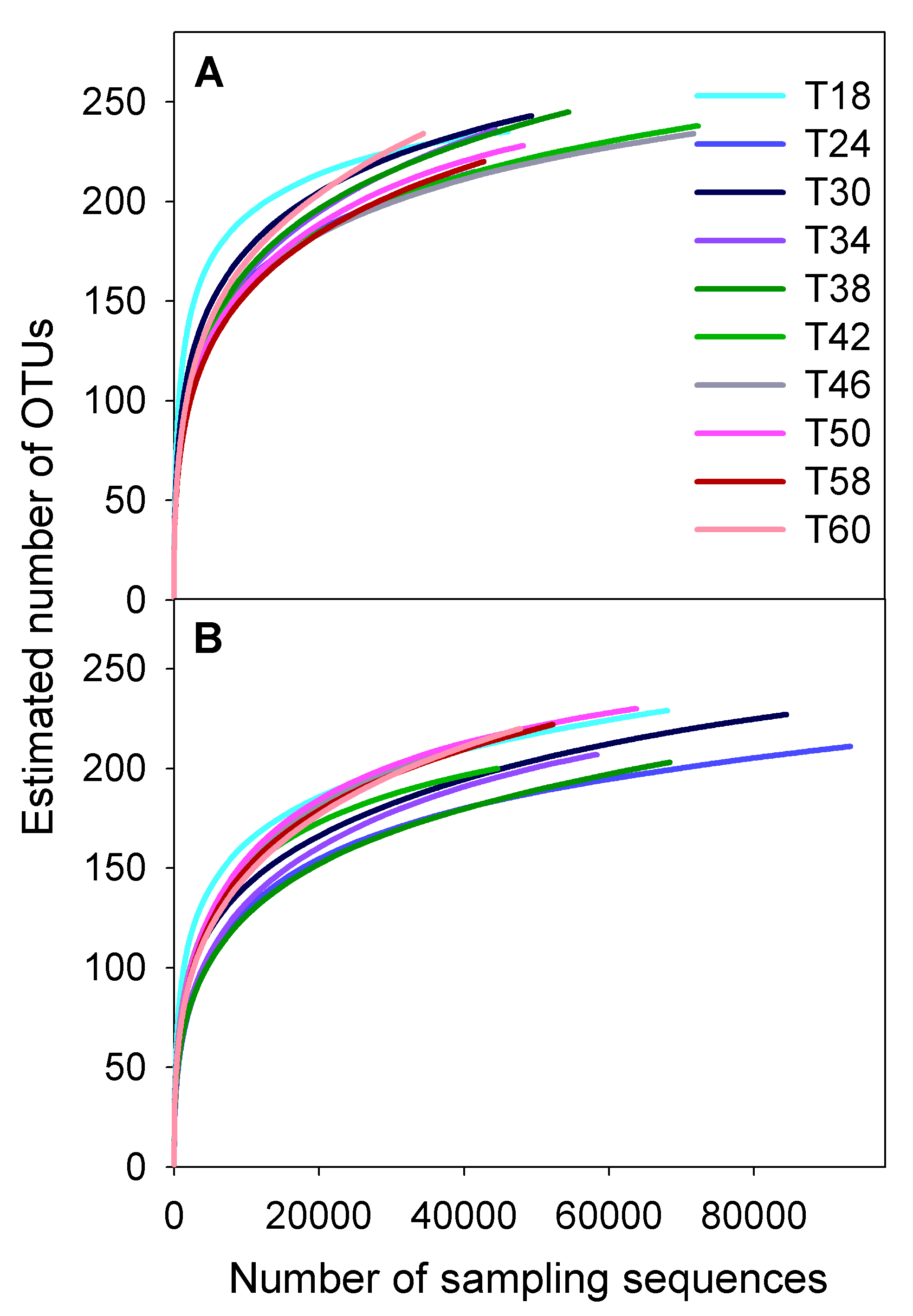

Supplement: S1 Fig — Individual rarefaction curves for each time-series sample taken from the MAnD (A) or TAnD (B) digesters. (TIF) [file pone.0181395.s001.tif]

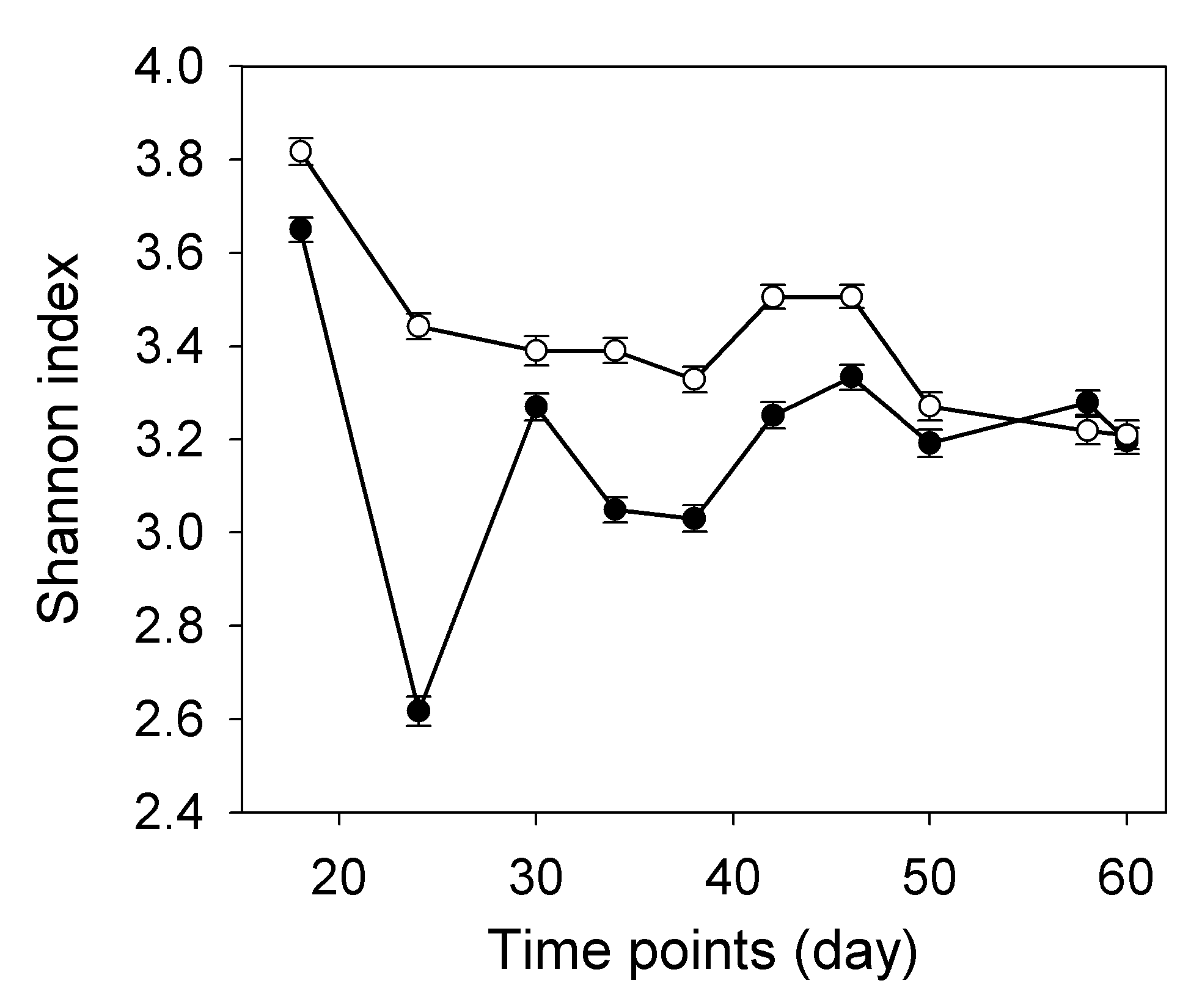

Supplement: S2 Fig — Diversity of microbial communities was represented as white or black circles for mesophilic (○) or thermophilic (●) conditions. The larger Shannon index represents a higher level of microbial diversity. (TIF) [file pone.0181395.s002.tif]
