# Supplementary material for: Inferring microbial interactions in thermophilic and mesophilic anaerobic digestion of hog waste
Source: PLoS One. 2017 Jul 21;12(7):e0181395. doi: 10.1371/journal.pone.0181395 (PMC5521784; doi:10.1371/journal.pone.0181395)
Supplement: S1 Table — (PDF) [file pone.0181395.s003.pdf]

S1 Table. Indicators of anaerobic digestion before or after day 60. SD means standard deviation. RCOD or RTS is the removal rate of COD or TS. MPR measures the amount of methane production where M or SE refers the statistical average or standard error of MPRs. CH_4% indicates the amount of MPR over that of the total gas production.

	**Before 60th day**		**After 60th day**	
	**37°C**	**55°C**	**37°C**	**55°C**
**SD(RCOD)**	8.63	8.21	1.67	1.20
**SD(RTS)**	3.48	5.05	1.24	1.23
**SD(CH_4%)**	4.91	10.02	1.41	1.16
**MPR (M±SE)**	0.13±0.02	0.24±0.04	0.36±0.03	0.56±0.02
