# Supplementary material for: Inferring microbial interactions in thermophilic and mesophilic anaerobic digestion of hog waste
Source: PLoS One. 2017 Jul 21;12(7):e0181395. doi: 10.1371/journal.pone.0181395 (PMC5521784; doi:10.1371/journal.pone.0181395)
Supplement: S2 Table — (PDF) [file pone.0181395.s004.pdf]

S2 Table. Differentially abundant families from each NBRP group under mesophilic (MAnD) or thermophilic (TAnD) conditions. The value is shown as % of total sequence in each sample. H, L or R indicates an OTU with high, low, or rare microbial abundance.

Taxonomy	Seeds	Manure	37°C	55°C	Diff	N_{genus}
Clostridia_unclassified	**0.18^{L}**	**0.02^{R}**	**0.37^{L}**	**10.11^{H}**	**UP**	**1**
Hydrogenophilaceae	0.51^{L}	-	0.02^{R}	0.65^{L}	UP	2
Peptococcaceae_1	<0.01^{R}	0.07^{R}	<0.01^{R}	0.03^{R}	UP	5
Clostridiales_unclassified	1.16^{H}	1.46^{H}	0.56^{L}	1.73^{H}	UP	1
Clostridiales_Incertae_Sedis_III	-	-	0.02^{R}	0.24^{L}	UP	1
Promicromonosporaceae	-	-	0.10^{L}	0.46^{L}	UP	1
Thermotogales_unclassified	-	-	0.02 ^{R}	1.81^{H}	UP	1
Atribacteria_unclassified	0.12^{L}	-	0.467^{L}	0.11^{L}	DN	1
Bacteroidetes_unclassified	13.69^{H}	1.26^{H}	16.14^{H}	3.32^{H}	DN	1
Burkholderiales_incertae_sedis	<0.01^{R}	-	0.40^{L}	0.18^{L}	DN	2
Chloroflexi_unclassified	0.70^{L}	<0.01^{R}	0.08^{R}	0.03^{R}	DN	1
Clostridiales_Incertae_Sedis_XIII	<0.01^{R}	-	0.17^{L}	0.06^{R}	DN	3
Deltaproteobacteria_unclassified	0.44^{L}	0.03^{R}	0.08^{R}	0.01^{R}	DN	1
Selenomonadales_unclassified	0.17^{L}	0.01^{R}	0.07^{R}	<0.01^{R}	DN	1
Planctomycetaceae	0.01^{R}	0.01^{R}	0.17^{L}	0.04^{R}	DN	2
