# Supplementary material for: Inferring microbial interactions in thermophilic and mesophilic anaerobic digestion of hog waste
Source: PLoS One. 2017 Jul 21;12(7):e0181395. doi: 10.1371/journal.pone.0181395 (PMC5521784; doi:10.1371/journal.pone.0181395)
Supplement: S4 Table — (PDF) [file pone.0181395.s006.pdf]

S4 Table. Interaction strength, complementarity and competition index under the mesophilic condition.

**Source family**	**Target family**	**Interaction strength**	**Complementarity**	**Competition**
p1: *Porphyromonadaceae*	p4: *Methanobacteriaceae*	0.083	0.156	0.265
p1: *Porphyromonadaceae*	p1-4: *Lachnospiraceae*	0.080	0.532	0.590
p1: *Porphyromonadaceae*	p1-4: *Prevotellaceae*	0.070	0.150	0.774
p3: *Erysipelotrichaceae*	p4: *Methanobacteriaceae*	0.057	0.087	0.382
p1: *Porphyromonadaceae*	p3: *Erysipelotrichaceae*	0.055	0.155	0.581
p3: *Erysipelotrichaceae*	p4: *Pseudomonadaceae*	0.054	0.005	0.349
p1: *Porphyromonadaceae*	p3: *Peptostreptococcaceae*	0.046	0.179	0.457
p1: *Porphyromonadaceae*	p1,2: *Clostridiacea*e_1	0.041	0.241	0.574
p1-4: *Ruminococcaceae*	p4: *Pseudomonadaceae*	0.034	0.019	0.415
p1-4: *Lachnospiraceae*	p4: *Pseudomonadaceae*	0.029	0.005	0.381
p3: *Erysipelotrichaceae*	p1-4: *Prevotellaceae*	0.028	0.247	0.491
p3: *Peptostreptococcaceae*	p4: *Pseudomonadaceae*	0.027	0.010	0.429
p4: *Pseudomonadaceae*	p1-4: *Prevotellaceae*	~0	0.002	0.396
p3: *Erysipelotrichaceae*	p1-4: *Ruminococcaceae*	-0.079	0.069	0.588
p1: *Porphyromonadaceae*	p1-4: *Ruminococcaceae*	-0.068	0.162	0.492
p1-4: *Lachnospiraceae*	p3: *Erysipelotrichaceae*	-0.049	0.175	0.532
p3: *Erysipelotrichaceae*	p4: *Enterobacteriaceae*	-0.041	0.072	0.500
p3: *Erysipelotrichaceae*	p1: *Bacteroidaceae*	-0.040	0.060	0.486
p1-4: *Prevotellaceae*	p3: *Erysipelotrichaceae*	-0.039	0.247	0.419
p1-4: *Lachnospiraceae*	p1: *Porphyromonadaceae*	-0.037	0.532	0.493
p3: *Erysipelotrichaceae*	p1: *Porphyromonadaceae*	-0.033	0.155	0.493
p1-4: *Prevotellaceae*	p1-4: *Ruminococcaceae*	-0.029	0.046	0.441
p3: *Peptostreptococcaceae*	p1: *Porphyromonadaceae*	-0.027	0.179	0.438
